# Identification and Characterization of Infectious Pathogens Associated with Mass Mortalities of Pacific Oyster (*Crassostrea gigas*) Cultured in Northern China

**DOI:** 10.3390/biology12060759

**Published:** 2023-05-23

**Authors:** Xiang Zhang, Bo-Wen Huang, Yu-Dong Zheng, Lu-Sheng Xin, Wen-Bo Chen, Tao Yu, Chen Li, Chong-Ming Wang, Chang-Ming Bai

**Affiliations:** 1 Key Laboratory of Maricultural Organism Disease Control, Ministry of Agriculture, Qingdao Key Laboratory of Mariculture Epidemiology and Biosecurity, Yellow Sea Fisheries Research Institute, Chinese Academy of Fishery Sciences, Qingdao 266071, China; zhxiang1997@126.com (X.Z.); huangbw@ysfri.ac.cn (B.-W.H.); zhengyudong032@163.com (Y.-D.Z.); xinls@ysfri.ac.cn (L.-S.X.);; 2 Laboratory for Marine Fisheries Science and Food Production Processes, Qingdao National Laboratory for Marine Science and Technology, Qingdao 266237, China; 3 Dalian Modern Agricultural Production Development Service Center, Dalian 116023, China; 4 Changdao Enhancement and Experiment Station, Chinese Academy of Fishery Sciences, Yantai 265800, China

**Keywords:** *Vibrio natriegens*, *Vibrio alginolyticus*, OsHV-1, pathogenicity, histopathology

## Abstract

**Simple Summary:**

The history of oyster culture consists of a succession of developmental phases using different species, followed by collapses associated with infectious diseases. The Pacific oyster (*Crassostrea gigas*), native to the northwest Pacific Ocean, was characterized by wide adaptability, and translocated to support major aquaculture industries in many countries around the world. A deadly herpesvirus, Ostreid herpesvirus-1 (OsHV-1), has hit the superior oyster since 1991. OsHV-1 infection, combined with subsequent bacteraemia by opportunistic bacteria, has brought heavy losses to the main production regions of the Pacific oyster. Because of the considerable economic importance of Pacific oysters, there is a wealth of information available on diseases that affect them around the world. Little comprehensive information is available about the epidemiology status of major disease-causing agents in China. On the other hand, the scale and yields of Pacific oyster increased rapidly in recent years, as a result of the introduction and popularization of triploid oysters. In the present study, a comprehensive survey of potential pathogens associated with mortality events of Pacific oysters was carried out. Our results highlight the potential risks of OsHV-1, *Vibrio natriegens*, and *Vibrio alginolyticus* to the aquaculture industry of the Pacific oyster in China.

**Abstract:**

The Pacific oyster (*Crassostrea gigas*) aquaculture industry increased rapidly in China with the introduction and promotion of triploid oysters in recent years. Mass mortalities affecting different life stages of Pacific oysters emerged periodically in several important production areas of Northern China. During 2020 and 2021, we conducted a passive two-year investigation of infectious pathogens linked to mass mortality. Ostreid herpesvirus-1 (OsHV-1) was detected to be associated with mass mortalities of hatchery larvae, but not juveniles and adults in the open sea. Protozoan parasites, such as *Marteilia* spp., *Perkinsus* spp. and *Bonamia* spp. were not detected. Bacterial isolation and identification revealed that *Vibrio natriegens* and *Vibrio alginolyticus* were the most frequently (9 out of 13) identified two dominant bacteria associated with mass mortalities. *Pseudoalteromonas* spp. was identified as the dominant bacteria in three mortality events that occurred during the cold season. Further bacteriological analysis was conducted on two representative isolates of *V. natriegens* and *V. alginolyticus*, designated as CgA1-1 and CgA1-2. Multisequence analysis (MLSA) showed that CgA1-1 and CgA1-2 were closely related to each other and nested within the *Harveyi* clade. Bacteriological investigation revealed faster growth, and more remarkable haemolytic activity and siderophore production capacity at 25 °C than at 15 °C for both CgA1-1 and CgA1-2. The accumulative mortalities of experimental immersion infections were also higher at 25 °C (90% and 63.33%) than at 15 °C (43.33% and 33.33%) using both CgA1-1 and CgA1-2, respectively. Similar clinical and pathological features were identified in samples collected during both naturally and experimentally occurring mortalities, such as thin visceral mass, discolouration, and connective tissue and digestive tube lesions. The results presented here highlight the potential risk of OsHV-1 to hatchery production of larvae, and the pathogenic role of *V. natriegens* and *V. alginolyticus* during mass mortalities of all life stages of Pacific oysters in Northern China.

## 1. Introduction

The Pacific oyster (*Crassostrea gigas*) is an economically and ecologically important species endemic to the waters of China, Korea, Japan, and Russia in the northwest Pacific Ocean [1]. Because of its rapid growth potential and wide adaptability to environmental conditions, the Pacific oyster has been introduced to many parts of the world as a supplemental aquaculture species for a decline of the local native oyster species [2]. In China, the Pacific oyster aquaculture production has increased from 0.81 million tonnes in 2000 to 1.46 million tonnes in 2018, with a growth rate of 3.32% per annum [3]. Beyond China, the Pacific oyster aquaculture production has been nearly stagnant from the mid-1980s [4], which fluctuates around 0.7 million tonnes, or even dropped to less than 0.6 million tonnes for several years [5]. China is responsible for most of the increased production of Pacific oyster aquaculture in recent decades [3,4]. The rapid increase in China could be attributed to a suite of technological advancements, such as artificial seeding in hatcheries, and longline aquaculture in the open sea [6]. More importantly, in recent years, the introduction and promotion of triploid oysters has stimulated more capital to be invested in the production and depuration of Pacific oysters in China [7]. Now, the Pacific oyster aquaculture industry has greatly commercialized nationwide in Northern China, with larval culture hatcheries concentrated along the coastline of LaiZhou, Shandong Province, and sold out to farmers for growing to market size. Before marketing, a short period (several weeks to months) for final fattening in bays with rich microalgae feed was always required. Therefore, long-distance transfer of live oysters was common during Pacific oyster production [6], which may contribute to the spread of mollusk pathogens [8].

High rates of Pacific oyster mortality (50–90%) could be traced back to 1927, which is shortly after the introduction of intensive hanging culture in Japan [9]. High water temperature and high salinity correlated with the spawning season, and pathogenic microorganisms were postulated to be responsible for the mortality [9]. After the war in 1945, the Pacific oyster was transplanted from its native range and established for large-scale commercial culture in Australia, America, and Europe. Similar epidemics with mortality rates ranging from 20 to 100% were reported periodically in many regions of the world where the species is cultivated [10]. Intensive studies have been carried out to investigate the causative factors involved in the syndrome [11,12,13,14]. A strictly pathological explanation has not been defined, though collective evidence suggested that multiplex intrinsic and extrinsic factors and their interactions were responsible for the phenomenon [15,16,17].

In recent decades, significant losses of oyster production have been reported more frequently, some of which were linked to pathogenic microorganisms including virus, bacteria, and parasites [8,18,19]. The most notorious mollusk parasites, *Perkinsus* spp., members of the Phylum Haplosporidia, and members of the Phylum Paramyxea, have caused serious local disruption to the complete collapse of indigenous oyster aquaculture industries in Europe and the USA [20]. Following that, *C. gigas* was then introduced successfully as a supplement to local indigenous oyster species. In 1991, mass mortalities of hatchery-reared Pacific oyster larvae were firstly reported to be associated with Ostreid herpesvirus-1 (OsHV-1) infection in France and New Zealand [21,22]. Subsequently, similar epidemics associated with OsHV-1 were reported in Mexico, Spain, and the USA [23,24,25]. In 2008, a new OsHV-1 variant (called μVar) emerged and outgrew the reference variant in France [26]. OsHV-1 μVar differs from the reference genotype at open reading frames (ORFs) 4 and 43, which primarily infects juvenile oysters (<12 months), and results in more serious mortality episodes [26]. OsHV-1 μVar has been subsequently identified as the primary etiological agent of Pacific oyster mortality syndrome (POMS) in a dozen countries [27]. Bacterial agents, especially members of the genus *Vibrio*, have also been linked to some cases of mass mortalities, which were capable of affecting all life stages of Pacific oysters [28,29,30]. More recently, it has also been proven that opportunistic bacteria, especially the strains assigned to the genus *Vibrio*, could act synergistically with OsHV-1 to contribute to the pathogenesis of mass mortality disease in Pacific oysters [31,32].

Increased mass mortalities of Pacific oysters were noticed in northern China in recent years, especially in the summer season. In the present study, we collected samples from mortality cases reported in multiple sites during 2020 and 2021. Multiple diagnosis methods, including molecular detection, histological investigation, bacteria isolation, and identification, were employed for the identification of potential etiologies. The key bacteriological features of the two identified pathogenic bacteria, *Vibrio alginolyticus* and *Vibrio natriegens*, were examined. The pathogenicity of the two *Vibrio* species was determined by experimental infection under controlled conditions.

## 2. Materials and Methods

### 2.1. Sample Collection and Preprocessing

**Figure 1:**
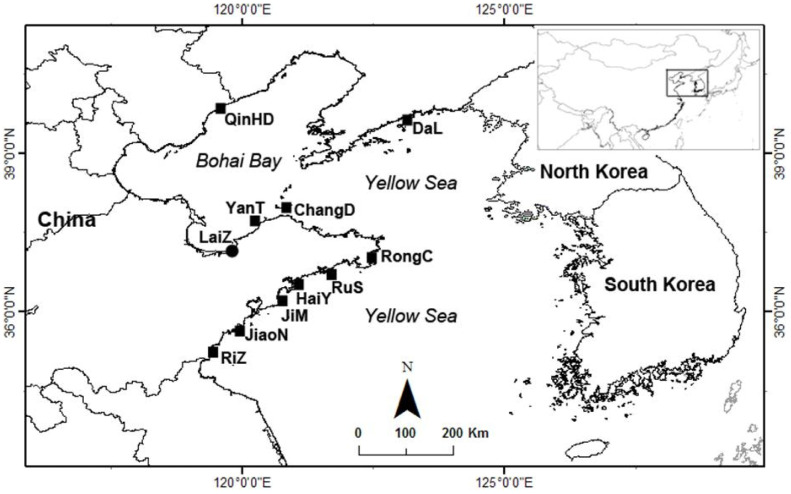
Sampling sites in northern China where the survey was conducted. ■ Indicate farms; ● indicate hatcheries.

Abnormal mortalities of Pacific oysters in Northern China were identified and reported by farmers according to their experiences. A total of eighteen batches of samples were collected from ten open sea areas and three hatcheries located in LaiZhou (Figure 1). Samples were collected and transported to the lab according to professional advice [33]. Macroscopic examinations were performed to record the shell abnormalities and fouling organisms. The juvenile and adult oysters were measured from bill to hinge, and opened for further investigation of gross abnormalities of viscera mass, such as predators, visible parasites, and physiological abnormalities. The larval samples were transferred to a petri dish with sterilized seawater at room temperature, and investigated under an inverted microscope.

### 2.2. DNA Extraction and qPCR Screening of Known Pathogens

A total of approximately 30 mg of targeted tissues (mantle and gills) of juvenile/adult oysters or larvae were collected from each sample for DNA extraction. The larvae samples were firstly ground using sterile pellet pestles to destroy the valves and expose soft tissues. The total DNA of each sample was extracted using the TIANamp Marine Animals DNA Kit (TIANGEN) according to the manufacturer’s instructions.

qPCR primer sequences and protocols for detection of OsHV-1, *Perkinsus* spp., *Marteilia* spp., and *Bonamia* spp. are listed in Table 1. Each sample was tested in duplicate, and the sample was recorded as positive if both replicates were amplified. For each run, total DNA from healthy oysters was used as a negative control; standard curves were generated using gradient dilutions of plasmids containing the targeted sequences of specific primers, which were synthesized by Sangon Biotech (Shanghai, China) Co., Ltd.

### 2.3. Bacteria Isolation and Identification

Only sample batches maintained at about 0–8 °C, and which arrived in the lab within 48 h after sampling, were subjected to bacterial isolation. For each batch, moribund individuals with weak responses to external stimuli were selected as priority for analysis. The external valves of the selected individuals were sprayed with 70% ethanol, then opened in sterile conditions and held on ice. The surfaces of targeted tissues (mantle, gill filament, and adductor muscles) were firstly wiped with 70% ethanol to remove the attached bacteria. Then, a mixture of about 50 mg tissues was dissected and placed in a 1.5 mL centrifuge tube using separate sterile scalpel blades and forceps for each oyster. The dissected samples of the juvenile and adult were rinsed with sterile seawater to remove alcohol and bacteria from the surface of the samples, and ground in 500 μL of sterile seawater with a sterile pellet-pestle (Tiangen Biotech Co, Beijing, China; No. OSE-Y50) for 1 min on average. For larval samples, they were rinsed using 500 μL of sterile seawater and ground as described above. The preparation of tissue homogenate was performed on ice before plate plating. Then, the homogenates of all samples were mixed, and 50 µL of supernatant was used to coat 2216E medium plates (Solarbio, NO. LA0340). The plates were incubated for 24–48 h at 28 °C, and observed for bacterial growth.

Colonies were identified according to their sizes, shapes, and colours on the plates. The numbers of colonies of each type for each sample were counted, which were used for identification of dominant types for each batch sample. The dominant types refer to the ones constituting at least 30% of all colonies. A representative colony of each dominant type was taken by a sterile loop and stroked onto new plates for purification. The purified isolates were then incubated in 1 mL 2216E liquid medium (Solarbio, NO. LA0341) at 28 °C overnight. A volume of 600 µL liquid cultures containing 40% glycerol were stored at −80 °C.

Bacterial DNA was extracted using the E.Z.N.A. ^®^Bacterial DNA Kit (Omega Bio-Tek Co., Guangdong, China; No. D3350-01) following the manufacturer’s instructions. The preliminary identification of isolated bacteria was performed using the primers 27F and 1492R to amplify 16S rDNA (Table 1). Sequencing was performed by Sangon Biotech Co., Ltd. (Shanghai, China).

**Figure 2:**
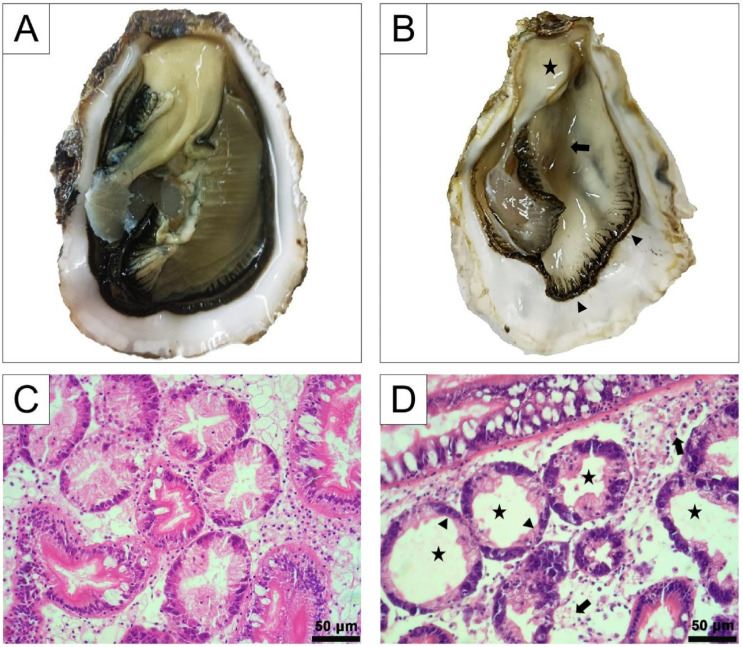
Clinical and histopathological characteristics of oysters infected with CgA1-1 and CgA1-2. (**A**) Healthy Pacific oyster. (**B**) Diseased Pacific oyster characterized by extreme emaciation. Arrowheads mark the thinned and shrunken mantle, the black arrow marks the atrophy of the gill, and the star marks the emaciated visceral mass. (**C**) Digestive gland of healthy Pacific oyster. (**D**) Digestive gland of diseased Pacific oyster. Black arrows mark the connective tissue necrosis and haemocyte infiltration, stars mark the enlarged and vacuolated digestive tubules, and arrowheads mark the sloughing of epithelial cells of digestive tubules. Scale bars in C and D = 50 μm (400× magnification).

In addition, two dominant pathogenic bacteria (*V. alginolyticus* and *V. natriegens*) associated with characteristic syndromes (Figure 2A,B) were selected for further study. Amplification and sequencing of housekeeping genes atpA (ATP synthase alpha subunit gene), mreB (rod shaping protein B subunit gene), pyrH (uridylate kinase gene), recA (recombinase A gene), rpoA (RNA polymerase alpha subunit gene), rpoD (RNA polymerase σ70 factor), and gyrB (DNA gyrase subunit B gene) were performed (primers used are shown in Table 1), as previously described, to further confirm their species [43]. The concatenated sequences of atpA-mreB-pyrH-recA-rpoA-rpoD-gyrB from the isolates and other reported Vibrio strains were aligned using the software Clustal X ver. 2.0 [44]. The phylogenetic tree was constructed using the neighbor-joining (NJ) method, and evaluated using bootstrap analysis with 1000 replicates. Phylogenetic analysis was carried out with tools embedded in MEGA ver. 7.0 [45], and visualized in iTOL ver. 6.1.1. “https://itol.embl.de/ (accessed on 1 September 2022)”.

### 2.4. Histopathology Analysis

For histopathologic analysis, cross-sections of oyster tissues were made to include most of the organs, including the digestive gland, gonad, intestine, gills, and adductor muscle, then fixed immediately in Davidson’s alcohol formalin–acetic acid fixative (DAFA) for 24–36 h. The fixed tissues were then dehydrated in an ascending ethanol series, cleared with two changes of xylene (1 h each), and immersed in liquid paraffin. After mounting in paraffin blocks, the tissue blocks were cut into 3–5 μm thick sections using a rotary microtome. The tissue sections were stained with hematoxylin and eosin (H&E) solution, then examined for the presence of parasites and pathological alterations.

### 2.5. Morphological and Cultural Characteristic Analysis

After incubating CgA1-1 and CgA1-2 in 2216E at 28 °C for 12 h, the bacterial culture went through static settlement for 20 min, the precipitate was washed twice with sterile seawater, and then concentrated 10-fold with sterile seawater. For electron microscopic observation, 10 μL of the sample was placed on the grid. The grid was stained with 2% phosphotungstic acid at neutral pH and stored at room temperature before imaging. The morphological structures of CgA1-1 and CgA1-2 were observed by transmission electron microscope (HT7700, Hitachi, Japan) with 6000× magnification. The remaining pure bacteria cultures were plastered on Vibrio selective agar (thiosulfate-citrate-bile salts-sucrose agar, TCBS), and plates were incubated at 28 °C for 24 h to investigate the colony morphology under microscope.

The strains CgA1-1 and CgA1-2 were inoculated on 2216E agar plates, the volume of inoculant being 10 μL, and then incubated at 28 °C for 24 h. A colony of each strain was then randomly selected and inoculated into 2216E liquid medium, and cultured at 28 °C until the OD_600_ value reached about 0.5. After that, 50 μL bacterial solution was inoculated in 50 mL 2216E liquid medium and cultured at different temperatures (15 ± 0.5 °C, 25 ± 0.5 °C) for 24 h. The absorbance value was measured at OD_600_ every two hours.

### 2.6. Haemolytic Activity and Chrome Azurol S Production Analysis

Overnight cultures of CgA1-1 and CgA1-2 were spotted on sheep blood agar plates, which were separated equally and incubated at 15 °C and 25 °C for 4 days. Haemolytic activity was assessed by visual inspection and measurement of the haemolytic zone.

Siderophore production was determined by the Chrome azurol S (CAS) method, as described by Schwyn and Neilands [46]. Overnight cultures of CgA1-1 and CgA1-2 were spotted on CAS agar medium, and incubated at 15 °C and 25 °C, respectively, for 6 days. Siderophore production was qualitatively observed by visual inspection and measurement of the orange-yellow halos around the colonies.

### 2.7. Experimental Infection

Oysters (measured 33.6–42.3 mm) without abnormal mortality were obtained from Jimo, China. Before the artificial infection experiments, the oysters were temporarily housed in tanks at 15 ± 0.5 °C or 25 ± 0.5 °C for two weeks. Oysters were fed daily with microalgae, and the water was changed once a week. The pacific oysters acclimated at different temperatures were randomly divided into four groups for each strain of CgA1-1 and CgA1-2, including 15 °C-challenged and control groups, 25 °C-challenged and control groups (60 pacific oysters were equally separated and cultured in three 50 L tanks for each group as repeats). Pacific oysters in the challenged group were immersed in strains CgA1-1 and CgA1-2, both with a final concentration of 1 × 10^5^ CFU/mL, while no bacteria were added to the control group. Mortality in each group was recorded at 12 h intervals for over 20 days, and both dead and moribund individuals were sampled. For each experimental group, 6 moribund samples were selected for bacterial isolation, identification, and histopathological investigation. For each negative control group, 3 individuals were randomly selected for histopathological investigation at the end of the experimental infection.

## 3. Results

### 3.1. Pathological Characteristics of Diseased Pacific Oysters

A total of eighteen batches of samples were collected, as shown in Table 2. Biofouling organisms such as barnacles, sponges, and blue mussels were occasionally observed, which may negatively affect the growth of the oysters. There were usually no characteristic gross abnormalities in the remaining populations after mass mortalities, except thinning, edema, erosions, and colour alteration of visceral mass were detected in some cases (Figure 2B). However, these clinical symptoms were always inconsistent among individuals, which could provide little information during the diagnosis. H&E-stained pathological sections of the clinically asymptomatic individuals always showed histopathological alterations. Different degrees of tissue lesions and accompanied haemocyte infiltrations were frequently observed in the connective tissue around the alimentary tract (intestine and stomach) (Figure 2D), and occasionally in the mantle. Sloughing of epithelial cells of digestive tubules into tubule lumens, and deterioration and vacuolization of the digestive tubules were also evident in thinner oysters (Figure 2D). No apparent pathology was observed in gonad and adductor muscle. The prodromal signs of diseased larvae were always a reduction of motility and feed intake, and an extension of rudimentary foot or velum.

qPCR screening of the known viral and parasite pathogens revealed that all tests were negative, except for OsHV-1 detection, in the five samples of larvae collected in April 2020. The viral DNA loads of the five samples ranged from 4.37 × 10^4^ to 3.82 × 10^5^ copies per ng of DNA, with an average of 1.79 × 10^5^ copies per ng of DNA.

### 3.2. Isolation and Identification of Bacteria from Diseased Pacific Oysters

Due to transportation and storage conditions, moribunds of 13 mortality cases were qualified for bacterial isolation. A total of 171 bacterial colonies were isolated. The 16S rDNA sequence analysis revealed that these colonies consisted of 55.56% *Vibrio* spp., 24.78% *Pseudoalteromonas* spp., 4.68% *Photobacterium* spp., 4.42% *Psychrobacter* spp., and the other genera comprising less than 3%. Bacteria that accounted for more than 30% of each batch were considered dominant, and are listed in Table 2. *V. alginolyticus* and *V. natriegens* were the two most frequently encountered dominant strains. The survey demonstrated that *V. alginolyticus* and *V. natriegens* acted as the dominant bacteria species in 9 out of the 13 investigated mortality cases, most of which were outbreaks in summer. *V. natriegens* was only identified as the dominant species in the summer season. Two isolates (CgA1-1 and CgA1-2) involved in summer mortality of Pacific oysters, with the most prevalent symptoms, were selected for further analysis.

**Figure 3:**
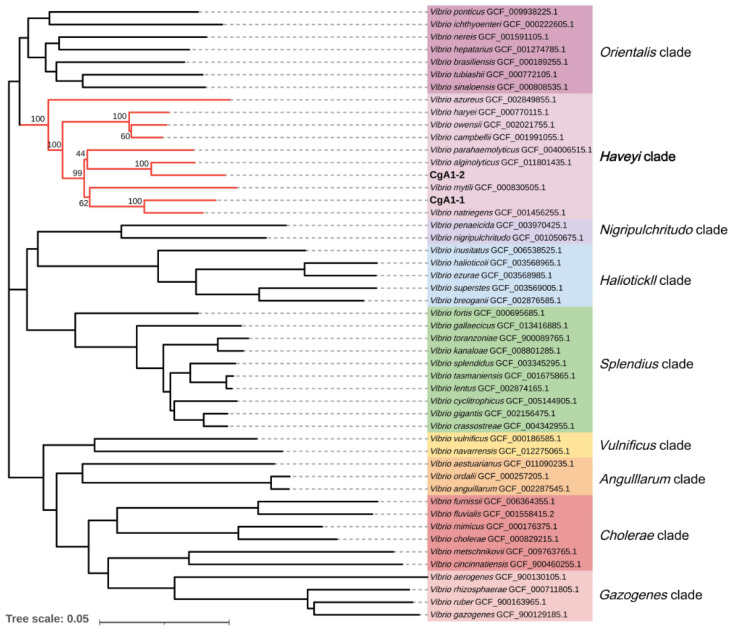
Phylogenetic tree of strain CgA1-1, CgA1-2, and representatives of *Vibrio* spp. The tree was constructed based on concatenated sequences of *atpA*, *mreB*, *pyrH*, *recA*, *rpoA*, *rpoD*, and *gyrB* genes using the NJ method. The coloured sectors indicate distinct *Vibrio* clades. The *Harveyi* clade is marked with a red line. The scale bar corresponds to 0.05 substitutions per site.

CgA1-1 and CgA1-2 were identified as *V. natriegens* and *V. alginolyticus*, respectively, by 16S rDNA sequence analysis. Sequencing and alignment of the seven housekeeping genes (*atpA*, *mreB*, *pyrH*, *recA*, *rpoA*, *rpoD*, and *gyrB*) of CgA1-1 and CgA1-2 resulted in a matrix of 6443 characters. Phylogenetic analysis was carried out using the concatenated sequences of the seven housekeeping genes of CgA1-1 and CgA1-2, and that of the other 50 representative *Vibrio* strains. The results firstly confirmed the taxonomic affiliation of CgA1-1 and CgA1-2 as inferred from 16S rDNA sequence analysis. The phylogenetic tree also showed that CgA1-1 and CgA1-2 form sister groups with *Vibrio mytili* and *Vibrio parahaemolyticus*, respectively, and the two groups further composed a sister clade that nested the *Harveyi* clade (Figure 3).

### 3.3. Phenotypic Characterization of CgA1-1 and CgA1-2

**Figure 4:**
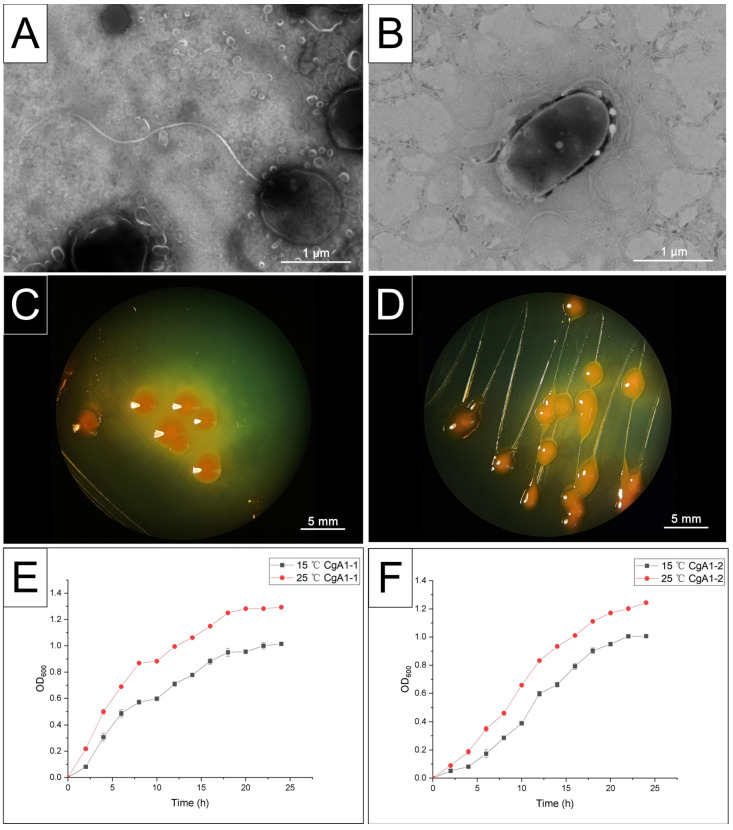
Morphological and growth characteristics of CgA1-1 and CgA1-2. (**A**) CgA1-1 cells were oval-shaped with one polar flagellum approximately 4.5 μm in length. (**B**) CgA1-2 cells were rod-shaped with lateral flagella observed at the surface of the cell. (**C**,**D**) Both CgA1-1 and CgA1-2 colonies were rounded on TCBS agar, with smooth edges and raised surfaces showing orange colour. (**E**,**F**) Growth dynamics of CgA1-1 and CgA1-2. Growth was assayed in 2216E medium by measuring OD_600_ for 24 h at 15 °C and 25 °C.

CgA1-1 was roughly spherical in shape, and approximately 1–1.2 μm in size. A single polar flagellum approximately 4.5 μm in length was observed by negative stain under electron microscope (Figure 4A). CgA1-2 was regularly rod-shaped of uniform size, approximately 1.5 μm long and 0.8 μm wide, with lateral flagella observed at the surface of the cell (Figure 4B). After incubation at 28 °C for 24 h on TCBS agar, both CgA1-1 and CgA1-2 colonies were round with smooth margins in shape, orange in colour, and nonmotile (Figure 4C,D). Growth of CgA1-1 and CgA1-2 at different temperatures (15 ± 0.5 °C, 25 ± 0.5 °C) showed significant differences, which grew faster when incubated at 25 °C (Figure 4E,F).

### 3.4. Haemolytic Activity and Siderophore Production Analysis

**Figure 5:**
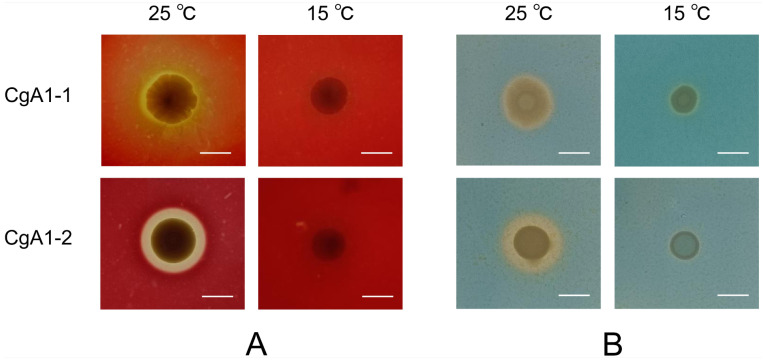
Haemolytic activity and siderophore production analysis. (**A**) Growth of strains CgA1-1 and CgA1-2 on sheep blood plate under high and low temperature, scale bar showing 1 cm. (**B**) Growth of strains CgA1-1 and CgA1-2 on CAS plate under high and low temperature, scale bar showing 1 cm.

Haemolytic activity and siderophore production were detected after spotting both CgA1-1 (*V. natriegens*) and CgA1-2 (*V*. *alginolyticus*) on blood agar plates and CAS agar plates, respectively (Figure 5). For each test, the signal was very weak at 15 °C, and evident at 25 °C.

### 3.5. Temperature Effects on the Pathogenicity of CgA1-1 and CgA1-2

**Figure 6:**
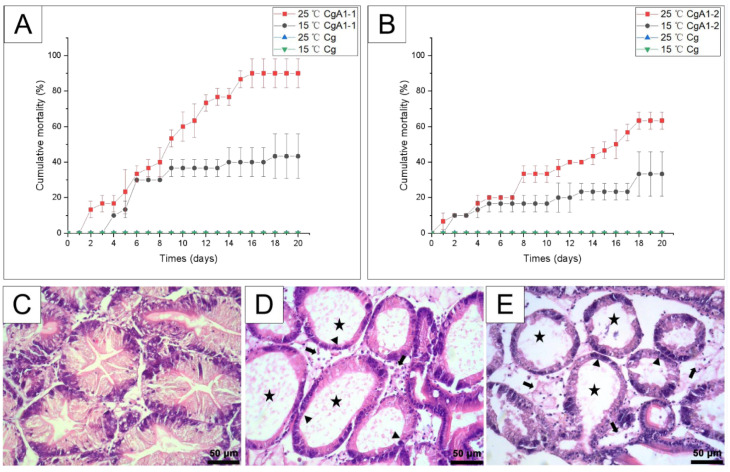
Cumulative mortality curve and histopathological features of experimentally infected Pacific oyster. (**A**,**B**) Cumulative mortality curve after exposure to CgA1-1 and CgA1-2 at 15 °C and 25 °C, respectively. (**C**) Histological features of oysters in the control group. (**D**,**E**) Histopathological features of oysters in experimental groups. Black arrows mark the connective tissue necrosis and haemocyte infiltration, stars mark the enlarged and vacuolated digestive tubules, and arrowheads mark the sloughing of epithelial cells of digestive tubules. Scale bar in (**C**–**E**) = 50 μm. H&E-stained histological sections viewed with 10× eyepiece and 40× objective.

To assess the pathogenicity of CgA1-1 and CgA1-2, and the effect of temperature, experimental infection was carried out at different temperatures (15 °C and 25 °C). The mortality rates of the experimental groups were significantly higher at 25 °C than at 15 °C for CgA1-1 (*χ*^2^ = 5.30, df = 1, *p* < 0.05), while only marginally significant for CgA1-2 (*χ*^2^ = 3.20, df = 1, *p* = 0.07). For the experimental groups at 25 °C, the mortality rate reached 90% and 63.33% at the end of the experimental infection for CgA1-1 and CgA1-2, respectively (Figure 6A). The mortality rates only reach 43.33% and 33.33% for experimental groups at 15 °C for CgA1-1 and CgA1-2, respectively (Figure 6B). The most evident gross clinical signs of moribund oysters were thinning and discolouration of visceral mass. *V. natriegens* and *V. alginolyticus* were isolated and identified as the dominant bacteria from corresponding experimental groups immersed with CgA1-1 and CgA1-2, respectively. Similar histopathological alterations were detected in moribund samples of all experimental groups (Figure 6D,E), which resembled those found in the naturally infected individuals, as described in Section 3.1. No clinical signs and mortality of oysters were observed in negative control groups (Figure 6C).

## 4. Discussion

With the development and promotion of triploid Pacific oysters in China, the aquaculture scale of Pacific oysters has expanded quickly in recent years. Mass mortalities of Pacific oysters were frequently reported in Northern China during the summer season, which is also a familiar phenomenon in other parts of the world [10,47]. At the same time, abnormal mortality cases were also found in the other seasons, affecting larval, juvenile, and adult oysters throughout the production process. There were more than ten infectious diseases of Pacific oysters, including those with viral, bacterial, protozoan, and metazoan etiologies [48]. Summer mortality syndrome [47,49,50], OsHV-1 infections [27], and Vibriosis have been identified as the main causes of disease outbreaks in recent decades [51,52], which led to the significant loss of production stocks around the world [24,53].

In the present study, most of the mortality events occurred during the warm season, suggesting that high temperatures may play a vital role in triggering mass mortality. *Vibrio* spp. have been frequently isolated from moribund Pacific oysters during the summer mortality events, which were suspected to be responsible for the mortality events [28,54,55]. For example, *Vibrio splendidus* and *Vibrio aestuarianus* were frequently identified in mortality events in France [51], *V. alginolyticus* was isolated in a mortality case reported in China [14,56], and *V. harveyi* was characterized in a mortality case that occurred in Australia [11]. In the present study, bacteria of the genus *Vibrio* were identified as the primary dominant strains in both hatcheries and open sea areas during 2020 and 2021 in China. Among them, *V. alginolyticus* and *V. natriegens* were the first two most predominant species associated with oyster mortality outbreaks. The abundance and pathogenicity of *V. alginolyticus* in Pacific oysters along some coastal areas in Shandong Province have also been proven in several other studies [14,57,58]. *V. alginolyticus* is also a widespread species of seawater Vibrio, which has been shown to cause disease in a variety of aquatic animals such as fish [59], shrimp [60], and shellfish [61]. *V. natriegens* has not been reported to be involved in mollusk mortality previously in China, while it was reported occasionally as the dominant bacterial species during summer mortality events in Pacific oysters in France [28]. However, the phenotypic and pathogenic properties of *V. natriegens* have not been characterized previously, according to our knowledge.

Environmental parameters, especially the seawater temperature, could dramatically affect the bacterial abundance and microbiome structure [54]. Warm (20–30 °C), mesohaline (<5–30) waters are the most hospitable for the growth of pathogenic *Vibrio* bacteria [62]. High temperatures in the summer season would drive the proliferation of *Vibrio* spp. in temperate waters [63]. In the present study, both *V. alginolyticus* and *V. natriegens* cultured under 25 °C outgrew their counterparts cultured under 15 °C. The virulence mechanisms of *V. alginolyticus* and *V. natriegens* are still undetermined. Several species of the *Harveyi* clade (including *V. alginolyticus*) have been reported to produce several common virulence factors, such as haemolysin and iron uptake system (*tonB*) [64]. Generating haemolytic molecules and producing siderophores have been proven to be two important phenotypes related to virulence properties [65,66,67]. In the present study, both *V. alginolyticus* and *V. natriegens* groups tested positive for haemolytic activity and siderophore production at 25 °C and 15 °C, with stronger signals being observed at 25 °C than at 15 °C in both Vibrio groups. Subsequent experimental infection using either *V. alginolyticus* or *V. natriegens* indicated that water temperature (25 °C and 15 °C) had a significant effect on the survival of Pacific oysters. These results, combined with the field observations, support the postulation that rising temperatures during the summer season may lead to an increase in Vibrio-associated illness in Pacific oysters [62,68].

The Pacific oyster is known for having been affected by a major worldwide epizootic disease associated with OsHV-1 μVar and related variants since 2008. Pacific oyster mortality syndrome (POMS) was created to refer to the mass mortalities due to Ostreid herpesvirus-1 (OsHV-1) [69], which has become panzootic, and represents a major threat to the Pacific oyster industry worldwide [27]. POMS is proven to be a polymicrobial (OsHV-1 variants and bacteria) and multifactorial (environmental and host factors) disease [17,70,71,72], and is now thought to be caused by an immune deficiency caused by OsHV-1 infection, thus allowing the conditional pathogen to take advantage of the situation, which results in oyster death [31]. The OsHV-1 infection (reference variant) was primarily limited to larval stages of *C. gigas* when the virus first emerged in 1991 [21,73]. Since 2008, OsHV-1 µVar and related variants have emerged and become the predominant variants of OsHV-1 worldwide [26,53,74,75,76,77]. Since the first report of shellfish mortalities associated with OsHV-1 in China in 1997, viral infection and the associated mass mortality have been reported in several bivalve species, such as *Chlamys farreri* [78,79], *C. gigas* [80,81], and *Scapharca broughtonii* [82,83]. The present study and previous unpublished epidemiological investigations indicate that no cases of mass mortality of juvenile or adult Pacific oysters were associated with OsHV-1 in China. POMS does not appear to be the main cause of Pacific oyster mortality in its natural populations in China. Similar situations were reported in the coastlines of Korea and Japan [84,85,86], the other two countries of the natural distribution range of Pacific oysters.

Pacific oysters are susceptible to a wide range of diseases caused by parasites, such as *Marteilioides chungmuensis* [87], *Perkinsus* spp. [88,89], *Haplosporidium* spp. [90], and *Mikrocytos mackini* [91]. The susceptibility of Pacific oyster to *Marteilia refringens*, *Bonamia ostreae*, and *Bonamia exitiosa* is still undetermined [92,93]. The occurrence and distribution patterns of parasites and associated diseases in Pacific oyster were not well characterized in China. In the present study, the presence of *Perkinsus* spp., *M. refringens*, *B. ostreae*, and *B. exitiosa* was screened by both histopathological and qPCR methods. No parasite or apparent infection was detected in these samples, which indicated that its effect on oyster populations was probably none or limited.

## 5. Conclusions

In summary, we carried out a two-year passive survey of mass mortalities associated with Pacific oysters. Although reporting bias and neglect of mortality cases might exist, it is meaningful to act as an alert and extent of mass mortality outbreaks that occurred in Pacific oysters. OsHV-1 infections were only detected during larvae production in hatcheries at low incidence. Considering the intensive production model of larvae and densely distributed hatcheries, the viral pathogen still poses a huge threat to the aquaculture industry of Pacific oysters. *V. natriegens* (CgA1-1) and *V. alginolyticus* (CgA1-2) were identified as potential pathogens that are closely associated with mass mortalities of Pacific oysters of different life stages in Northern China. Phenotypic assays and pathogenic analyses of CgA1-1 and CgA1-2 indicated that temperature plays a key role during the growth and virulence of the two Vibrios. Long-term sea-surface temperature increases during the summer season will lead to an increase in Vibrio-associated illness of Pacific oysters in Northern China. The results suggested that *V. alginolyticus* and *V. natriegens* are two widespread and dominant bacteria in oyster-farming areas, and should be monitored as a priority in the future.

## Figures and Tables

**Table 1:** Sequences and references of the primers and probes employed in the present study.

Primer Name	Product	Primer Sequence (5′–3′)	References
OsHV-1			[34]
BF		GTCGCATCTTTGGATTTAACAA	
B4		ACTGGGATCCGACTGACAAC	
BP		FAM-TGCCCCTGTCATCTTGAGGTATAGACAATC-BHQ	
*Perkinsus* spp.			[35]
PERK-F		TCCGTGAACCAGTAGAAATCTCAAC	
PERK-R		GGAAGAAGAGCGACACTGATATGTA	
PERK-P		FAM-CCCTTTGTGCAGTATGC-MGB	
*Marteili* spp. and *Bonamia* spp.			[36]
Mar-18S-F		ACGATCAAAGTGAGCTCGTG	
Mar-18S-R		CAGTTCCCTCACCCCTGAT	
Mar18S-IN		FAM-GCATGGAATCGTGGAACGGG-BHQ	
Bosp2-18S-F		CAGGATGCCCTTAGATGCTC	
Bosp2-18S-R		GTACAAAGGGCAGGGACGTA	
Bosp2-18S-IN		HEX-TTGACCCGGCTTGACAAGGC-BHQ	
*Vibrio* housekeeping gene			
27F	16S rDNA	AGAGTTTGATCCTGGCTCAG	[37]
1492R		GGTTACCTTGTTACGACTT	
atpA-F	ATP synthase alpha subunit	CTDAATTCHACNGAAATYAGYG	[38]
atpA-R		TTACCARGWYTGGGTTGC	
mreB-F	Rod shaping protein B subunit	ACTTCGTGGCATGTTTTC	[39]
mreB-R		CCGTGCATATCGATCATTTC	
pyrH-F	Uridylate kinase	ATGASNACBAAYCCWAAACC	[40]
pyrH-R		GTRAABGCNGMYARRTCCA	
recA-F	Recombinase A	TGARAARCARTTYGGTAAAGG	[40]
recA-R		TCRCCNTTRTAGCTRTACC	
rpoA-F	RNA polymerase alpha subunit	ATGCAGGGTTCTGTDACAG	[41]
rpoA-R		GHGGCCARTTTTCHARRCGC	
rpoD-F	Factor σ70 RNA polymerase	ACGACTGACCCGGTACGCATGTAYATGMGNGARATGGGNACNGT	[40]
rpoD-R		ATAGAAATAACCAGACGTAAGTTNGCYTCNACCATYTCYTTYT	
gyrB-F	Gyrase B subunit	GAAGTCATCATGACCGTTCTGCAYGCNGGNGGNAARTTYRA	[42]
gyrB-R		AGCAGGGTACGGATGTGCGAGCCRTCNACRTCNGCRTCNGYCAT	

**Table 2:** Mortality events and dominant bacteria identified over 2 years in Northern China.

Sampling Date	Sampling Site	Month Age	Temp. (°C)	No. of Samples ^a^	Shell Height (mm)	Mortality (%)	Dominant Bacteria
2020.03	HaiY	10	6.8	20 (3)	90.5 ± 8.6	60	*Vibrio alginolyticus*
2020.05	LaiZ ^c^	larvae	25.3	5 tubes (0)	/	>80	/
2020.05	RiZ	12	18.6	42 (3)	118.6 ± 9.5	40	*Vibrio alginolyticus*
2020.07	RongC	3	19.8	26 (0)	35.9 ± 3.3	70	/
2020.08	JiM ^b^	2	27.2	30 (0)	14.6 ± 2.1	>70	/
2020.09	DaL	5	24.6	20 (3)	55.3 ± 5.1	80	*Vibrio natriegens*
2020.09	DaL	4	25.1	8 (2)	53.7 ± 4.8	85	*Vibrio natriegens* *Vibrio alginolyticus*
2020.12	ChangD ^b^	8	9.5	24 (3)	88.9 ± 7.8	40–50	*Pseudoalteromonas nigrifaciens*
2020.12	ChangD	8	9.4	32 (3)	94.3 ± 8.3	50	*Pseudoalteromonas elyakovii*
2021.04	LaiZ	larvae	25.2	3 tubes (0)	/	80	/
2021.05	LaiZ	larvae	25	13 tubes (3)	/	50	*Pseudoalteromonas piratica* *Vibrio alginolyticus*
2021.05	LaiZ	larvae	26	7 tubes (1)	/	70–80	*Vibrio harveyi*
2021.06	RuS	13	12.3	12 (3)	122.6 ± 9.6	50	*Vibrio aestuarianus*
2021.07	YanT	3	23.5	30 (3)	35.4 ± 3.5	75	*Vibrio natriegens* *Vibrio alginolyticus*
2021.08	DaL	4	22.1	30 (0)	56.8 ± 5.1	70	/
2021.08	QinHD	4	28.3	20 (3)	52.3 ± 4.4	60–70	*Vibrio natriegens* *Vibrio alginolyticus*
2021.10	JiaoN	6	18.2	30 (5)	64.3 ± 5.2	50–60	*Vibrio fortis* *Vibrio alginolyticus*
2021.10	JiaoN ^b^	6	18.3	30 (5)	56.4 ± 4.7	50	*Vibrio alginolyticus*

^a^ Numbers in parentheses represent the number of samples used for bacterial isolation. ^b^ The three batches marked are diploid oysters, the rest are triploid oysters. ^c^ The one batch of samples tested positive for OsHV-1 infection.

## Data Availability

The authors confirm that the data supporting the findings of this study are available within the article.
